# The *Plasmodium* Class XIV Myosin, MyoB, Has a Distinct Subcellular Location in Invasive and Motile Stages of the Malaria Parasite and an Unusual Light Chain*

**DOI:** 10.1074/jbc.M115.637694

**Published:** 2015-03-23

**Authors:** Noor A. Yusuf, Judith L. Green, Richard J. Wall, Ellen Knuepfer, Robert W. Moon, Christina Schulte-Huxel, Rebecca R. Stanway, Stephen R. Martin, Steven A. Howell, Christopher H. Douse, Ernesto Cota, Edward W. Tate, Rita Tewari, Anthony A. Holder

**Affiliations:** From the Divisions of ‡ Parasitology,; ‖ Physical Biochemistry, and; ** Molecular Structure, MRC National Institute for Medical Research, London NW7 1AA, United Kingdom,; the § School of Life Sciences, Queens Medical Centre, University of Nottingham, Nottingham NG2 7UH, United Kingdom,; the ¶ Institute of Cell Biology, University of Bern, CH-3012 Bern, Switzerland, and; the ‡‡ Institute of Chemical Biology, Imperial College London, South Kensington, London SW7 2AZ, United Kingdom

**Keywords:** Invasion, Malaria, Molecular Motor, Myosin, Parasite, Peptide Interaction, Plasmodium, Myosin Light Chain

## Abstract

Myosin B (MyoB) is one of the two short class XIV myosins encoded in the *Plasmodium* genome. Class XIV myosins are characterized by a catalytic “head,” a modified “neck,” and the absence of a “tail” region. Myosin A (MyoA), the other class XIV myosin in *Plasmodium*, has been established as a component of the glideosome complex important in motility and cell invasion, but MyoB is not well characterized. We analyzed the properties of MyoB using three parasite species as follows: *Plasmodium falciparum, Plasmodium berghei,* and *Plasmodium knowlesi*. MyoB is expressed in all invasive stages (merozoites, ookinetes, and sporozoites) of the life cycle, and the protein is found in a discrete apical location in these polarized cells. In *P. falciparum,* MyoB is synthesized very late in schizogony/merogony, and its location in merozoites is distinct from, and anterior to, that of a range of known proteins present in the rhoptries, rhoptry neck or micronemes. Unlike MyoA, MyoB is not associated with glideosome complex proteins, including the MyoA light chain, myosin A tail domain-interacting protein (MTIP). A unique MyoB light chain (MLC-B) was identified that contains a calmodulin-like domain at the C terminus and an extended N-terminal region. MLC-B localizes to the same extreme apical pole in the cell as MyoB, and the two proteins form a complex. We propose that MLC-B is a MyoB-specific light chain, and for the short class XIV myosins that lack a tail region, the atypical myosin light chains may fulfill that role.

## Teaser

**Background:** Malaria parasites possess two unusual class XIV myosins, myosin A that drives gliding motility and myosin B that is uncharacterized.

**Results:** Myosin B is located at the extreme apical end of motile and invasive parasites, binding a very large and unusual light chain.

**Conclusion:** Myosin B differs substantially from myosin A in location and function.

**Significance:** An unusual myosin and its light chain extend the known diversity of these families.

## Introduction

The malaria parasite genome contains six genes that encode myosins (1, 2). Two of these genes code for short class XIV myosins, which are restricted to Apicomplexan parasites and other members of the Alveolata. These myosins differ from most others in that, although they have the highly conserved “head” region that binds actin and hydrolyzes ATP, and the “neck” region that acts as the lever arm and contains IQ motifs to which myosin light chains bind, they have no “tail” region. The tail normally allows oligomerization, cargo binding, and determines the subcellular location of the myosin (3). Of the two short class XIV myosins encoded by *Plasmodium falciparum*, myosin (Myo)^5^A is the most studied and has been implicated as providing the power for gliding motility and cell invasion as part of the glideosome complex (4, 5). The glideosome complex is found between the parasite plasma membrane and the underlying inner membrane complex. It also contains myosin A tail domain-interacting protein (MTIP), which is a myosin light chain, together with glideosome-associated proteins (GAP) 45, GAP50, and GAP40 (5). Despite its name, MTIP binds to the C-terminal part of MyoA, which should strictly be called a neck rather than tail domain (6–10). It is the membrane association of MTIP and the GAPs that confers inner membrane complex localization upon MyoA (11–14). In contrast, the second short class XIV myosin, MyoB, has not been studied in detail, and its properties are largely unknown. There has been one report of the location of *P. falciparum* MyoB, which described it as localized within merozoites (15).

In this study, we have tagged MyoB with GFP and HA and examined its expression and cellular localization both within the asexual blood stage development of *P. falciparum* and *Plasmodium knowlesi* and throughout the life cycle of *Plasmodium berghei*. We identify a new and unusual myosin light chain that binds to a region at the C terminus of MyoB through a calmodulin-like domain. The myosin light chain contains an extended N terminus when compared with other more typical light chains, and we propose this may be acting as a tail domain for MyoB. We also identify a unique apical localization for this complex in invasive stages of the parasite.

## EXPERIMENTAL PROCEDURES

### Ethics Statement for Animal Work

All animal work passed an ethical review process and was approved by the United Kingdom Home Office. Work was carried out in accordance with the United Kingdom Animals (Scientific Procedures) Act of 1986 and in compliance with European Directive 86/609/EEC for the protection of animals used for experimental purposes. The project license number is 40/3344. Six- to 8-week-old female Tuck-Ordinary (TO) outbred mice (Harlan) were used for all experiments.

### Parasite Genetic Modification

#### 

##### GFP Tagging of P. falciparum MyoB and MyoA

**FIGURE 1.**
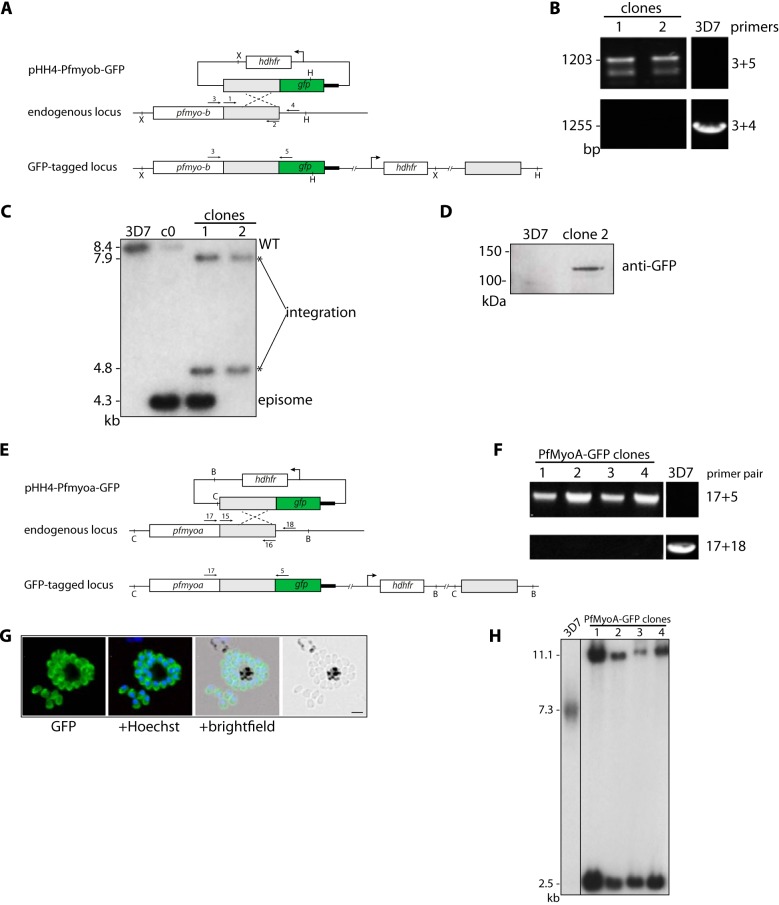
**Generation of PfMyoB-GFP and PfMyoA-GFP parasites.** *A,* schematic representation of the GFP-tagging of PfMyoB by single crossover homologous recombination into the *myoB* locus. The primers for PCR (*arrows 1* and *2*) and the Southern blot probe together with restriction sites are labeled. *X* = XbaI and *H* = HpaI. *B,* diagnostic PCR on genomic DNA showing integration of PfMyoB-GFP (*primers 3*+*5*) and wild type (*primers 3*+*4*). Two PfMyoBGFP clones were examined. *C,* Southern blot analysis of cloned PfMyoB-GFP parasites. Genomic DNA was digested with XbaI and HpaI restriction enzymes. A probe to the *myob* region of homology showed the following: PfMyoB-GFP cycle 0 (*c0*) shows the presence of wild-type (8.4 kb) and episome (4.3 kb) bands; 3D7 parasites only show the wild-type band. Clone 1 shows the expected bands for integration (7.9 and 4.8 kb), but also for episome, suggesting concatamer insertion. Clone 2 shows only bands for integration and was therefore used in all subsequent experiments. *D,* Western blot. Extracts of late stage schizonts from 3D7 and PfMyoB-GFP clone 2 parasites were immunoblotted wth an anti-GFP antibody. MyoB-GFP protein of ∼120 kDa was detected in clone 2. *E,* schematic representation of the GFP tagging of MyoA by single crossover homologous recombination into the *myoA* locus, with primers for PCR (*arrows* with primer pair 15 and 16) and Southern blot probe and restriction sites labeled. *C* = ClaI and *B* = BsrFI. *F,* diagnostic PCR on genomic DNA showing integration of PfMyoA-GFP (*primers 17*+*5*) and wild type (*primers 17*+*18*). Four PfMyoA-GFP-expressing clones were examined. *G,* PfMyoA-GFP-expressing merozoites as viewed by live fluorescence microscopy. GFP was detected by green fluorescence, and the nuclei (*blue*) were labeled with Hoechst dye prior to microscopic analysis; the GFP signal is distributed to the parasite periphery. *Scale bar,* 2 μm. *H,* Southern blot analysis of cloned PfMyoA-GFP-expressing parasites. Genomic DNA was digested with ClaI and BsrFI. When probed with the *myoa* region of homology, all clones showed the expected two integration bands at 11.1 and 2.5 kb. 3D7 is the wild-type control and shows a band of the expected size (7.3 kb).

A 1069-bp sequence from the 3′ end of the *P. falciparum* 3D7 *myoB* ORF without the stop codon was amplified from genomic DNA by PCR using primer pairs 1 and 2 (all primers used are listed in Table 1) and cloned between the XmaI/AvrII sites of the pHH4-GFP plasmid^6^ generating the construct pHH4-PfMyoB-GFP in which the targeting fragment was placed upstream of the GFP ORF followed by the *P. berghei dhfr* 3′UTR. The correct sequence of the plasmid was confirmed by Sanger sequencing (Beckman Genomics). After transfection of ring stage parasites with 100 μg of plasmid DNA, 2.5 nm WR99210 was added, and the parasites were cultured continuously under drug selection for ∼3 weeks (designated cycle 0). The transfected parasites were then grown for 3 weeks without drug selection, to allow loss of episomal DNA followed by a further week of growth under WR99210 selection (cycle 1). The cycling was repeated one more time. Cultures were checked after each cycle for integration of the construct into the genome by diagnostic PCR using primer pairs 3 and 4 to detect integration and primer pairs 3 and 5 to detect the unmodified locus and for GFP expression by fluorescence microscopy. After confirmation of correct integration, parasite lines were cloned by limiting dilution. A scheme for parasite integration, diagnostic PCR, and Southern blot is shown in Fig. 1.

**TABLE 1:** **Oligonucleotide primers used in this study** Primers used in this study are listed with nonhomologous sequences in lowercase, restriction enzyme sites in lowercase italic, and nonhomologous sequences introduced to generate a unique restriction site in bold, lowercase font.

ID	Oligonucleotide sequence	Description
1	*cccggg*GGACATCCTTTCGCCTAACATTTTGAAA	PfMyoB-F
2	*cctagg*TTCGTGTTCCTTAATATATTTATATTTCCTAAAATATGC	PfMyoB-R
3	CATAACGGAAAGTTTTGTAATTAAGCATACTGTAAGTGATG	diagPfMyoB-F
4	GGATGACCAGAAAGTTGTAACTTTGGCAAATTTATTC	diagPfMyoB-R
5	CTCCAGTGAAAAGTTCTTCTCC	GFP-R
6	ttttaa*cccggg*GCGTGATATCCATTTTGGAGG	PkMyoB-F
7	cccatt*cctagg*CTTCAGCTGTGCAATGTACTTATG	PkMyoB-R
8	GATCATCGCTGAGTCTGTGAAG	diagPkMyoB-F
9	GTGTGTAGTGAACGAGTACAACG	diagPkMyoB-R
10	CCTTTACCGCGGTCAAGCGTAATCTGGAACGTCGTAAGGGTAGCCCATGG	HA-R
11	cccc*ggtacc*ATACCGTAAGTGATGTAACATATACTATTAC	PbMyoB-F
12	cccc*gggccc*TTCGAGCTCTTTCATATGCTTATATTTTCG	PbMyoB-R
13	CGAAGCATGCAAAATATGCTTCTACAAAG	diagPbMyoB-F
14	ACGCTGAACTTGTGGCCG	diagPbMyoB-R
15	*cccggg*CAAGAATTGAACCAGAAGGAGGATTTAAAAC	PfMyoA-F
16	*cctagg*TTGAGCTACCATTTTTTTTCTTATATGAGCTTGTA	PfMyoA-R
17	GGGAAATATTAATTAAGGTAACTGTTGCTGGAGGAAC	diagPfMyoA-F
18	CACTCATAGATGCAACATGTAAGTATACAAATAAATATGGTATGTAG	diagPfMyoA-R
19	*cccggg*AGGAGACGTTAAGTTTGGAGAA	PfMLC-B-F
20	*cctagg*TTCTTCGGTTCCATAAATCCA	PfMLC-B-R
21	ATATGTATGTATGCTATAATGGGTTAT	diagPfMLC-B-F
22	GGACAAAATGGAAAAGTTTTTTTTTAGACAATGTAGAG	diagPfMLC-B-R
23	*cccggg*TGTGAATCATCTGCAATACGGCAGGAA	PkMLC-B-F1
24	*cctagg*gtgtgtggt**acc**GACACGTCGAAGATACTCTCCCCGTTT	PkMLC-B-R1
25	**ggt**ACCCAGCTATACCATTCTCCCACAT	PkMLC-B-F2
26	*cctagg*CTCCCCTGTTCCTTGGATCCA	PkMLC-B-R2
27	gtca*ggatcc*GAAGAACAAGAATTATTAGAAGAA	PfMLC-B-CTDexpF
28	gtca*ctcgag*TTATTCTTCGGTTCCATAAATCCA	PfMLC-B-CTDexpR
29	GAAAATATAAGAGTAGTATCACAGGAAG	PbMyoB-RT-FW
30	CTATCATCAATTTCTGGTATAACCAC	PbMyoB-RT-RV
31	GTATTATTAATGAACCCACCGCT	Pbhsp70FW
32	GAAACATCAAATGTACCACCTCC	Pbhsp70RV
33	TTGATTCATGTTGGATTTGGCT	PbarginylFW
34	ATCCTTCTTTGCCCTTTCAG	PbarginylRV
35	gatc*ggatcc*AAAATTGAGGAATTGGAAATGAACATTAGTTATTGTG	PfMLC-B-CCexpF
36	gatc*ctcgag*TTACTCCTTATTTTTCTCGGATATCTTCTTGGT	PfMLC-B-CCexpR

A parasite line expressing MyoA-GFP was prepared in a similar fashion. A region of homology corresponding to the final 1057 bp of the *P. falciparum myoA* gene was amplified from genomic DNA using primers 15 and 16 and cloned into the pHH4-GFP plasmid exactly as described for *Pfmyob*. A scheme for integration and its analysis by Southern blot and diagnostic PCR using primer pairs 17 and 5 to detect integration and 17 and 18 to detect the unmodified locus is shown in Fig. 1, together with the pattern of GFP fluorescence in the parasite.

##### HA Tagging of P. knowlesi MyoB

**FIGURE 2.**
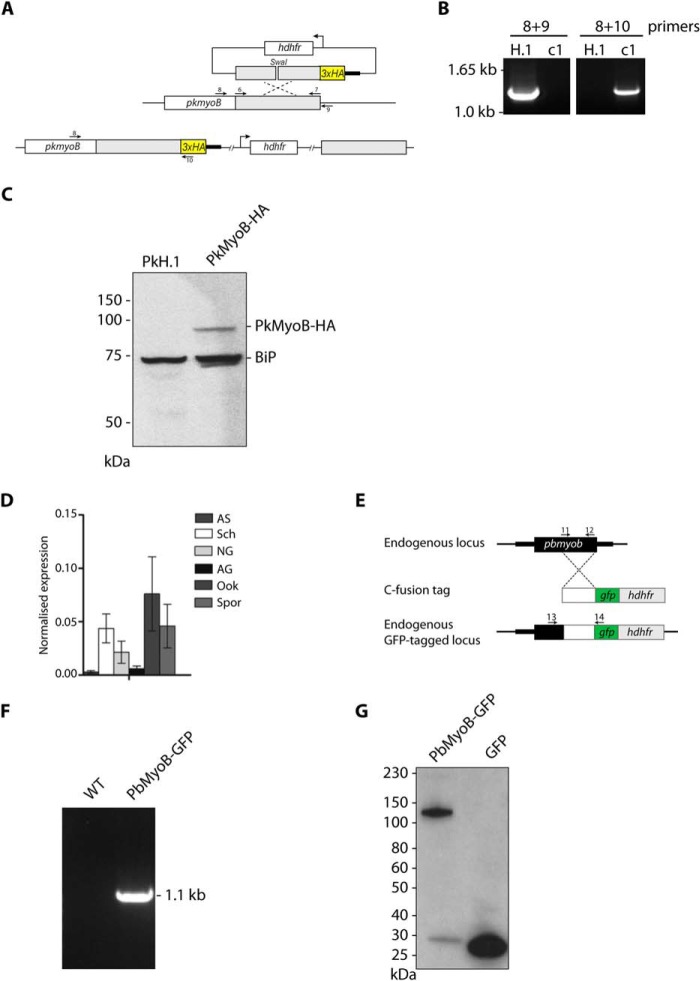
**Generation of PkMyoB-HA and PbMyoB-GFP and detection of *myob* throughout the *P. berghei* life cycle.** *A,* diagram representing C-terminal tagging of *Pkmyob* with a triple HA tag. Primers shown (*arrows*) were used to amplify the region of homology (6 and 7) for diagnostic PCR of WT parasite sequences (8 and 9) or parasites where integration had taken place (8 and 10). *B,* diagnostic PCR on genomic DNA showing integration of PkMyoB-HA into the *myob* locus in clone c1 with parental H.1 DNA as a control. *C,* Western blot with an anti-HA antibody on parental PkH.1 and PkMyoB-HA parasite lysates. In PkMyoB-HA parasites, a protein of ∼90 kDa is detected. The same blot was probed with an antibody against BiP to demonstrate equivalent sample loading. *D,* quantitative RT-PCR to show mRNA expression of *Pbmyob* during the parasite life cycle. Arginyl-tRNA synthetase and *hsp70* were used as endogenous controls for normalization. *Bars,* three biological replicates, each ± S.E. *AS,* all asexual blood stages; *Sch,* schizonts; *NG,* nonactivated gametocytes; *AG,* activated gametocytes; *Ook,* ookinetes; *Spz,* sporozoites. *E,* diagram representing C-terminal tagging of *Pb*MyoB with GFP by single homologous recombination into endogenous *myob* gene, showing primers 11 and 12 to amplify the region of homology, and primers 13 and 14 to detect integration. *F,* confirmation of integration by PCR of *Pbmyob-gfp* using primer pair 13 and 14. *G,* Western blot of extracts of parasites expressing *Pb*MyoB-GFP or GFP.

A 1316-bp DNA fragment corresponding to the 3′ end of the *PkmyoB* gene was amplified using primers 6 and 7 and cloned via XmaI/AvrII sites into the pHH4PK vector by having replaced the sequence coding for GFP with a sequence coding for a triple HA epitope tag. The vector was linearized using SwaI, and 10 μg of DNA was transfected as described previously (16). A scheme for construct integration and diagnostic PCR screening with primer pairs 8 and 10 to detect integration and 8 and 9 to detect the unmodified locus are shown in Fig. 2.

##### GFP Tagging of P. berghei MyoB

*P. berghei myoB* was modified by single homologous recombination to insert a sequence coding for a C-terminal GFP tag, using the p277 vector that contains a human *dhfr* selection cassette (Fig. 2*E*) (17). A 978-bp sequence of *myoB* starting 2.4 kb downstream of the ATG initiator codon and omitting the stop codon was amplified using primers 11 and 12 and inserted into the vector using KpnI and ApaI restriction sites. The construct was linearized using HincII and then used to transfect the *P. berghei* ANKA 2.34 line by electroporation (18). The electroporated parasites were mixed immediately with 150 μl of reticulocyte-rich blood, incubated at 37 °C for 30 min, and then injected intraperitoneally into naive mice. Pyrimethamine (Sigma) at 7 mg/ml was administered to the drinking water for 4 days to select resistant parasites. Mice were monitored for 10 days to detect parasites, and the drug selection was repeated after parasite passage to a second mouse. Pyrimethamine-resistant parasites were then analyzed with a diagnostic PCR using primers 13 and 14 to determine correct integration of the *gfp* sequence at the targeted locus (Fig. 2*F*), and a Western blot was performed to confirm the size of the GFP-tagged protein (Fig. 2*G*).

##### GFP Tagging of P. falciparum MLC-B

**FIGURE 3.**
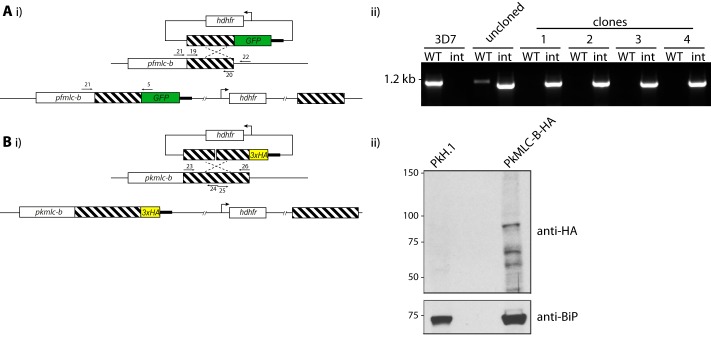
**Generation of PfMLC-B-GFP and PkMLC-B-HA parasites.** *A, panel i,* schematic representation of the GFP tagging of PfMLC-B by single crossover homologous recombination into the *pfmlc-b* locus. Primer pair 19 and 20 were used to amplify the region of homology. Primer pair 21 and 22 amplify from the wild-type locus, and pair 21 and 5 were used to detect integration of the plasmid into the target locus. *Panel ii,* diagnostic PCR shows integration at the *Pfmlc-b* locus for four independent clones. *B, panel i,* schematic representation of the HA tagging of PkMLC-B by single crossover homologous recombination into the *Pkmlc-b* locus. Primer pairs 23 and 24 and 25 and 26 were used to generate the region of homology (described under “Experimental Procedures”). *Panel ii,* Western blot probed with an anti-HA antibody detects a product of the expected size in parasites where integration has taken place. Molecular mass markers are indicated on the *left* in kDa.

A 1034-bp fragment of homology targeting the *mlc-b* locus (PF3D7_1118700) was amplified from 3D7 genomic DNA using the primer pair 19 and 20. The PCR fragment was inserted into pHH4-GFP between the XmaI and AvrII sites, verified by sequencing, and transfected into 3D7 parasites as above. WR99210-resistant parasites were cloned by limiting dilution. Parasites were screened for integration into the *mlc-b* locus by diagnostic PCR using primer pairs 21 and 5 to detect integration and 21 and 22 to detect the unmodified locus, as shown in Fig. 3*A*.

##### HA Tagging of P. knowlesi MLC-B

A 1198-bp region of homology representing 3739–4933 bp from *pkmlc-b* was cloned into the XmaI/AvrII sites of pHH4PK-HA vector using a two-step approach. A region of *pkmlc-b* from 3739 to 4308 bp was amplified from genomic DNA using the primer pair 23 and 24, where underlining denotes the introduced unique KpnI site. After cloning into pGEM-T Easy and sequence verification, this region was excised and cloned into the XmaI/AvrII sites of the pHH4PK-HA vector. A second region of *pkmlc-b* from 4309 to 4933 bp was amplified using the primer pair 25 and 26, where nonhomologous sequences were introduced to generate the KpnI site. After cloning into pGEM-T Easy and sequence verification, this region was excised and cloned into the KpnI/AvrII sites of the pHH4PK-*mlc-b*-HA vector generated in the previous step. This plasmid was linearized with AleI prior to transfection into *P. knowlesi* (Fig. 3*B*) (16).

##### Analysis of PbMyoB Gene Expression by Quantitative RT-PCR

Blood stage *P. berghei* parasites from infected mice (day 4 post-infection) were cultured *in vitro* (40 ml of RPMI 1640 medium, 8 ml of fetal bovine serum, 0.5 ml of penicillin and streptomycin per 0.5 ml of blood) for 24 h at 37 °C (with rotation at 100 rpm). The following day, schizont-infected cells were purified on a 60% v/v NycoDenz/PBS gradient (NycoDenz stock solution: 27.6% w/v NycoDenz in 5 mm Tris-HCl, 3 mm KCl, 0.3 mm EDTA, pH 7.2). The purification of gametocytes was based on a modified protocol described in Beetsma *et al.* (19). On day 4 post-infection, parasites were harvested, kept on ice to avoid premature activation, and separated from uninfected erythrocytes on a 48% (v/v) NycoDenz in coelenterazine loading buffer (CLB) gradient (CLB: PBS containing 20 mm HEPES, 20 mm glucose, 4 mm sodium bicarbonate, 1 mm EGTA, 0.1% w/v bovine serum albumin, pH 7.25). Gametocytes were harvested from the interface and washed twice in RPMI 1640 medium before activation of gamete formation in ookinete medium (20) for 30 min at 20 °C. For ookinete preparation, parasites from day 5 post-infected mice were placed in ookinete medium for 24 h at 20 °C; the parasites were then lysed in red blood cell lysis buffer for 30 min and purified on a 63% NycoDenz gradient (v/v in PBS). On day 14 post-feeding, guts were dissected and crushed in a loosely fitting homogenizer to release sporozoites, which were then quantified using a hemocytometer (20).

Total RNA was isolated from purified parasites using an RNeasy purification kit (Qiagen). cDNA was synthesized using an RNA-to-cDNA kit (Applied Biosystems) allowing quantification from 250 ng of total RNA. Each quantitative PCR consisted of 2 μl of cDNA, 5 μl of SYBR Green fast master mix (Applied Biosystems), 0.5 μl (500 nm) each of the forward and reverse primers, and 2 μl of diethyl pyrocarbonate-treated water. Where possible, one of the primer pairs was placed over an intron and together they amplified a region 70–200 bp long. Analysis was conducted using an Applied Biosystems 7500 fast machine with the following cycling conditions: 95 °C for 20 s followed by 40 cycles of 95 °C for 3 s; 60 °C for 30 s. Wild-type expression was determined using the Pfaffl method (21). The method used *hsp70* (PBANKA_081890) and arginyl-tRNA synthetase (PBANKA_143420) as reference genes. All primers used are listed in Table 1 (primers 29–34).

### Antibodies

Polyclonal rat anti-PfMyosin B antibodies were raised to a peptide of the sequence MVNKINELNNYFRINSTFINKSENE by Pepceuticals Ltd. according to their standard protocol. Rabbit anti-EBA175 and mouse anti-RAP1 (7H8/54) were obtained from the Malaria Research and Reference Reagent Resource Centre. Mouse anti-RON4 was a gift from Dr. J-F Dubremetz (University of Montpellier). Anti-α-tubulin mAb clone DM 1A (T9026, Sigma) was used to detect *P. falciparum* α-tubulin. Rabbit anti-GFP^6^ and rat anti-HA (monoclonal 3F10, Roche Applied Science) antibodies were used to detect tagged proteins. Species-specific AlexaFluor 488- and 594-conjugated secondary antibodies were obtained from Molecular Probes (Life Technologies, Inc.).

### Microscopy

#### 

##### Live Imaging of GFP-tagged Parasite Lines

*P. falciparum* and *P. knowlesi* parasite-infected red blood cells were prepared for live imaging as described in Ridzuan *et al.* (22) and viewed using an Axio Imager M1 microscope (Zeiss). *P. berghei* asexual blood stage infections in mice were initiated by intraperitoneal injection of parasite-infected blood. The asexual, gametocyte, and ookinete stages were observed using Hoechst 33342 dye in ookinete medium. To examine mosquito mid-gut infection, guts were dissected 14 days after feeding on infected blood and mounted under Vaseline-rimmed coverslips after staining with Hoechst dye for 10–15 min. On day 21 post-feeding, guts and salivary glands were dissected and crushed separately in a loosely fitted homogenizer to release sporozoites, which were then quantified using a hemocytometer and used for imaging (20). Microscopy analysis was performed using a Zeiss AxioImager M2 microscope (Carl Zeiss, Inc) fitted with an AxioCam ICc1 digital camera.

For *P. berghei* liver stage parasites, 60,000 HepG2 cells were seeded in glass-bottomed imaging dishes. Salivary glands of female *Anopheles stephensi* mosquitoes infected with *Pb*MyoB-GFP parasites were isolated and disrupted using a pestle. Sporozoites were pipetted gently onto seeded HepG2 cells and incubated at 37 °C in 5% CO_2_ in complete minimum Eagle's medium containing 2.5 μg/ml amphotericin B (PAA). Medium was changed 3 h after initial infection and once a day thereafter. For live cell imaging, Hoechst 33342 (Molecular Probes) was added to a final concentration of 1 μg/ml, and parasites were imaged 24, 48, and 55 h post-infection using an epi-fluorescence microscope (DMI600B from Leica).

##### Indirect Immunofluorescence Assay

Thin smears of parasites were fixed with 4% paraformaldehyde for 30 min at room temperature. The fixed cells were permeabilized with PBS containing 0.1% Triton X-100 for 5 min followed by blocking in 3% BSA (w/v) in PBS at 4 °C overnight. Primary antibodies were diluted as required; species-specific Alexa Fluor 488- and 594-conjugated secondary antibodies (Life Technologies, Inc.) were used to visualize primary antibody binding. All antibody dilutions were carried out with 3% BSA (w/v) in PBS. Slides were mounted for microscopic examination using Prolong Gold antifade reagent with DAPI (Life Technologies, Inc.). Slides were viewed using a Zeiss Axioplan 2 microscope, and images were captured using a Zeiss Axiocam MRc digital camera and AxioVision 4.8.2 software and prepared for publication using Adobe Photoshop.

To image stages of invasion of erythrocytes, tightly synchronized PfMyoB-GFP-expressing late stage schizonts were mixed with erythrocytes, and samples were taken after 2, 5, 8, 10, and 30 min and fixed in solution with 4% paraformaldehyde, 0.01% glutaraldehyde for 60 min at room temperature. Further processing was carried out as described previously (23).

### Preparation of Parasite Lysates, Immunoprecipitation of GFP Fusion Proteins, and Analysis of the Precipitates by Western Blotting and LC-MS/MS

#### 

##### Parasite Lysates

Schizonts from the MyoB-GFP, MyoA-GFP, MLC-B-GFP, and 3D7 parasite lines were first lysed in 0.15% (w/v) saponin in PBS and harvested by centrifugation, and the proteins were extracted using 10 cell pellet volumes of ice-cold lysis buffer (0.5% Nonidet P-40, 150 mm NaCl, 10 mm Tris-HCl, pH 7.5, 1× Complete protease inhibitors (Roche Applied Science)). Samples were cleared by centrifugation at 100,000 × *g* for 10 min. The detergent concentration in the samples was reduced to 0.2% by dilution with 150 mm NaCl, 10 mm Tris, pH 7.5, 1× Complete protease inhibitors.

##### Immunoprecipitation

Parasite lysates were pre-cleared by incubation with blocked agarose beads (Chromotek) for 1 h, and then the supernatant was incubated with GFP-Trap-agarose beads (Chromotek) for 2 h at 4 °C with end-over-end rotation. The beads were washed extensively with 150 mm NaCl, 10 mm Tris, pH 7.5, and then subjected to a further wash in 300 mm NaCl, 10 mm Tris, pH 7.5. Finally the beads were resuspended in 5 volumes of 2× SDS-PAGE sample buffer and boiled for 5 min prior to fractionation of the proteins by SDS-PAGE.

##### Western Blotting

Cell lysates were separated under reducing conditions on NuPAGE gels (Life Technologies, Inc.). Proteins were transferred to nitrocellulose and blocked in 5% w/v nonfat milk in PBS + 0.2% Tween 20. Primary antibodies were diluted in 5% w/v nonfat milk in PBS + 0.2% Tween 20. Species-specific horseradish peroxidase-conjugated secondary antibodies (Bio-Rad) were used, and the signal was developed using enhanced chemiluminescence (GE Healthcare). Blots were exposed to BiomaxMR film (Eastman Kodak), and images were prepared for publication using Adobe Photoshop.

##### Protein Identification by LC-MS/MS

Proteins were run 4 mm into a 10% NuPAGE BisTris gel (Life Technologies Inc.) and then excised using a clean scalpel blade. Proteins were reduced and alkylated prior to overnight trypsin digestion. The resulting digests were analyzed by LC-MS/MS using an Ultimate 3000 nanoRSLC HPLC, equipped with a 50-cm × 75-μm Acclaim Pepmap C18 column, coupled to an LTQ Orbitrap Velos Pro equipped with a Nanoflex electrospray source (all Thermo Scientific). A gradient of 6–32% acetonitrile, 0.1% formic acid over 48 min was used at a flow rate of 0.3 μl/min. The Orbitrap was operated in data-dependent acquisition mode with a survey scan at 60,000 resolution and up to the 10 most intense ions selected for MS/MS. Raw files were processed using Proteome Discoverer 1.3 (Thermo Scientific) with Mascot 2.4 (Matrix Science, UK) as the search engine against the appropriate protein database. A decoy database of reversed sequences was used to filter the results at a false detection rate of 1%. Proteins that were represented by three or more peptides in the MyoB-GFP or MLC-B-GFP datasets but were absent in the corresponding 3D7 dataset were included in further analyses.

##### Bioinformatic Analyses

Alignment of sequences was performed using a progressive alignment algorithm (24) within CLC Sequence Viewer 6 (CLC Bio). Phylogenetic tree construction was performed using CLC Sequence Viewer 6, using the neighbor joining method (25) and 100 replicates for bootstrap analysis. Coiled-coil prediction was performed using MARCOIL and Scorer 2.0 (26, 27). A structural model of PfMLC-B calmodulin (CaM)-like domain (amino acids 508–645) was constructed using Phyre 2 protein fold recognition server (28). Amino acids 508–645 were modeled on the CaM moiety of a GFP/M13/CaM fusion protein (Protein Data Bank code 3evr) with 97.6% confidence. Images were prepared using CCP4 Molecular Graphics software (29).

##### Cloning and Expression of Recombinant MLC-B Fragments

RNA was prepared from late *P. falciparum* schizonts (∼45 h post-invasion) using TRIzol (Life Technologies, Inc.), and cDNA was made from this using the reverse transcription system with oligo(dT) primers (Promega). The primer pair 27 and 28 was used to amplify the 3′ 471 bp of *mlc-b*, and primer pair 35 and 36 was used to amplify nucleotides 901–1227 from the cDNA. These fragments were cloned into the BamHI and XhoI sites of pGEX6P1 (GE Healthcare). The plasmids were transformed into BL21 Gold cells (Stratagene), and protein expression was induced by the addition of 1 mm isopropyl β-d-1-thiogalactopyranoside for 3 h at 37 °C for MLC-B CTD or 18 °C for MLC-B amino acid residues 301–409. The cell pellet was lysed overnight at 4 °C in 20 mm Tris-HCl, pH 8.0, 250 mm NaCl, 0.5 mm tris(2-carboxyethyl)phosphine (TCEP), and 2 mg/ml lysozyme (Sigma). The supernatant was incubated with glutathione-Sepharose 4b resin (GE Healthcare) for 2 h at 4 °C, followed by extensive washing with 20 mm Tris-HCl, pH 8.0, 250 mm NaCl, 0.5 mm TCEP. The beads were resuspended in 3 volumes 20 mm Tris-HCl, pH 8.0, 100 mm NaCl, 0.5 mm TCEP, 4 units/ml PreScission protease (GE Healthcare) and incubated, with mixing, overnight at 4 °C to cleave the MLC-B fragment from the GST moiety. The supernatant containing the cleaved MLC-B fragment was collected and concentrated using a Vivaspin20 concentrator with a 5-kDa molecular mass cutoff (Sartorius). For MLC-B CTD buffer exchange into PBS was carried out on a PD10 column (GE Healthcare).

### Biophysical Analysis of MLC-B CTD and Its Interaction with MyoB Neck Peptides

#### 

##### Peptides

Myosin B peptides ^763^ITSALIMKIKKKR^775^ and ^780^NIKNLQLAQAYFRKYKYIKEH^800^ were synthesized and purified as described elsewhere (30). The N termini of both peptides were acetylated, and the C termini were capped with an amide to remove the unnatural charge.

##### Far-UV CD

Far-UV CD spectra were recorded on a Jasco J-815 spectropolarimeter fitted with a cell holder thermostatted by a CDF-426S Peltier unit. To monitor peptide binding, CD measurements were made at a protein concentration of 9.1 μm PfMLC-B CTD (0.17 mg/ml) and 27 μm peptide (0.04 mg/ml Ile^763^ to Arg^775^, and 0.07 mg/ml Asn^780^ to His^800^) in 25 mm Tris-HCl, pH 8.0, 50 mm NaCl using fused silica cuvettes with 1-mm path length (Hellma, Jena, Germany). Far-UV CD spectra of PfMLC-B fragment 301–409 were obtained at a protein concentration of 11 μm (0.15 mg/ml). All spectra were recorded with 0.1 nm resolution and baseline-corrected by subtraction of the buffer spectrum. Secondary structure content was estimated using methods described by Sreerama and Woody (31).

##### Thermal Unfolding

Thermal unfolding curves were obtained by monitoring the ellipticity at 222 nm using 2-mm path length cuvettes and a heating rate of 1 °C/min over the temperature range 20–90 °C. The transition mid-point temperature was obtained by fitting data to the modified Gibbs-Helmholtz equation using in-house software as described elsewhere (32).

## RESULTS

### 

#### 

##### MyoB Is Located at the Extreme Apical End of Merozoites, Ookinetes, and Sporozoites, the Invasive Stages of the Malaria Parasite

To establish the subcellular location of MyoB we generated transgenic lines in three different parasites, *P. falciparum, P. knowlesi*, and *P. berghei*, fusing MyoB to either a GFP or a triple HA tag. *P. falciparum* and *P. knowlesi* were used to examine MyoB expression during the asexual blood stage, and *P. berghei* was used primarily to look at expression in the mosquito stages and in the liver stage of the life cycle.

**FIGURE 4.**
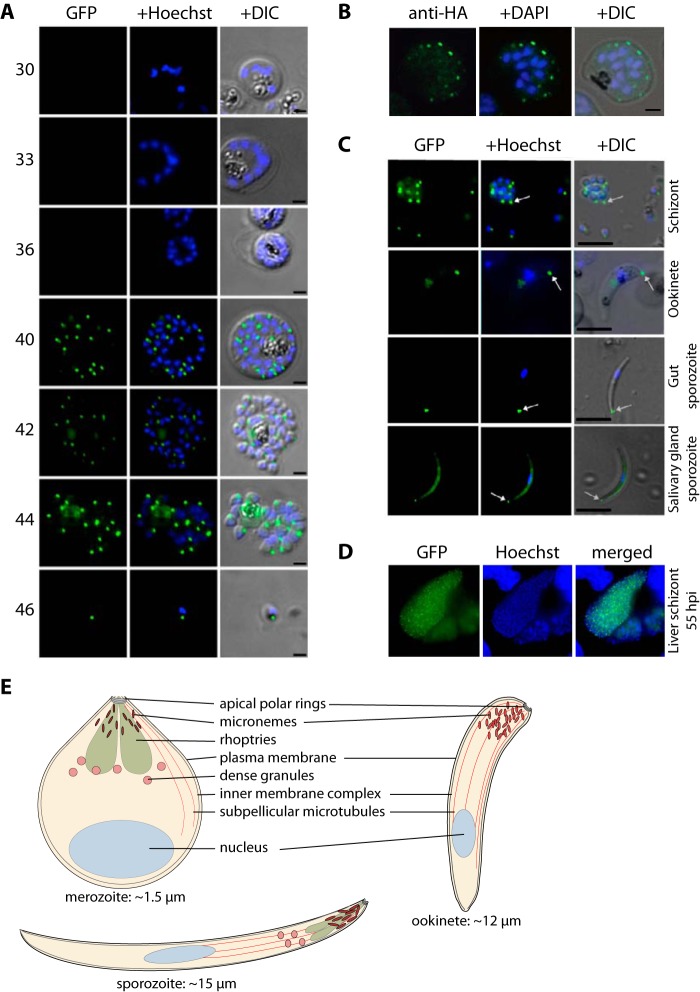
**MyoB subcellular location in asexual blood and mosquito stages.** *A,* microscopic analysis of live *P. falciparum* asexual blood stage parasites expressing GFP-tagged MyoB. Shown are blood stage parasites of increasing maturity from early schizogony (two to four nuclei; 30 h post-invasion) through to mature segmenter forms (44 h post-invasion) and free merozoites analyzed by fluorescence microscopy. GFP was detected by green fluorescence, and the nuclei (*blue*) were labeled with Hoechst dye prior to microscopic analysis. GFP fluorescence was not detected in trophozoites (data not shown) and early schizont stages, and was first apparent in mature multinucleate schizonts as a number of single dots (40 h post-invasion). Following cytokinesis, a single dot was present associated with each nucleus at what appeared to be the apical end of the cell. The images, merged with the differential interference contrast (*DIC*) image, are shown in the *right panel. Scale bar,* 2 μm. *B,* immunofluorescence of MyoB-HA in *P. knowlesi* schizonts. The epitope is detected using a specific antibody and an AlexaFluor 488 secondary antibody. Nuclei are labeled with DAPI; the *green*, *blue,* and differential interference contrast-merged images are also shown. *Scale bar,* 2 μm. *C,* expression of MyoB-GFP in the three invasive stages: schizonts (merozoites), ookinetes, and sporozoites of *P. berghei*. GFP is detected by green fluorescence, and nuclei (*blue*) were labeled with Hoechst dye. The merged and differential interference contrast images are also shown. The *white arrows* indicate expression of MyoB-GFP at the apical end of the parasites. *Scale bar,* 5 μm. *D,* expression of MyoB-GFP in liver stage schizonts of *P. berghei*, 55 h after invasion by a sporozoite. GFP was detected by green fluorescence, and the nuclei (*blue*) were labeled with Hoechst dye prior to microscopic analysis. Merged images are shown in the *right panel. Scale bar,* 10 μm. *E,* schematic showing the three invasive stages of *Plasmodium*, merozoite, ookinete, and sporozoite. The approximate length from anterior to posterior is shown.

For *P. falciparum,* we used single crossover homologous recombination to integrate sequences into the *myob* locus and express the endogenous MyoB with a C-terminal GFP. Correct integration was confirmed by Southern blotting, diagnostic PCR, and protein expression (Fig. 1). Live parasite microscopy was used to examine expression of MyoB-GFP during the parasite's asexual blood stage development in the red blood cell. Up until 38 h after invasion, when each schizont has 8–10 nuclei, there was no clear green fluorescence signal detected. MyoB-GFP expression as a distinct dot was first observed when each schizont had 10 or more nuclei, corresponding to ∼40 h post-invasion (Fig. 4*A*). This clear pattern persisted following cytokinesis and formation of mature segmented schizonts at 42–44 h after invasion and was also present in merozoites (46 h). These data indicate that MyoB-GFP is synthesized very late in schizont development, starting just before segmentation, and the protein is located in a single site within each merozoite, at the anterior end of the cell. This is also clearly apparent when looking at *P. knowlesi* schizonts where endogenous MyoB has been tagged with a triple HA tag at its C terminus (Fig. 2). Because of the larger size, smaller number, and ordered “apex-outward” arrangement of the *P. knowlesi* merozoites, MyoB-HA can be clearly identified at the apical end of the parasite (Fig. 4*B*), similar to the pattern seen for PfMyoB-GFP.

In *P. berghei*, MyoB was present in all of the invasive stages of the life cycle. Expression was detected by quantitative RT-PCR with mRNA prepared from six key stages of parasite development (Fig. 2*D*). MyoB expressed from the endogenous locus was tagged with GFP, using a single crossover recombination strategy (Fig. 2*E*), and integration of the GFP sequence was confirmed by diagnostic PCR (Fig. 2*F*). Protein expression in schizonts was confirmed by Western blotting (Fig. 2*G*). Protein expression and location within the cell were examined by live fluorescence microscopy. We detected MyoB-GFP as a distinct single dot in developing merozoites within schizonts, ookinetes, and sporozoites, with the fluorescence concentrated at the extreme apical end of the cell in each of these forms of the parasite (Fig. 4*C*). In salivary gland sporozoites, in addition to the bright apical spot of fluorescence, we also observed a more diffuse fluorescence within the body of the parasite. This was not observed in any other stages, and the significance, if any, of this is unknown. In addition, in mature liver-stage schizonts 55 h after invasion by sporozoites, individual merozoites displayed an apical dot localization for PbMyoB-GFP (Fig. 4*D*). The protein was absent in late schizonts (48 h after invasion; data not shown).

**FIGURE 5.**
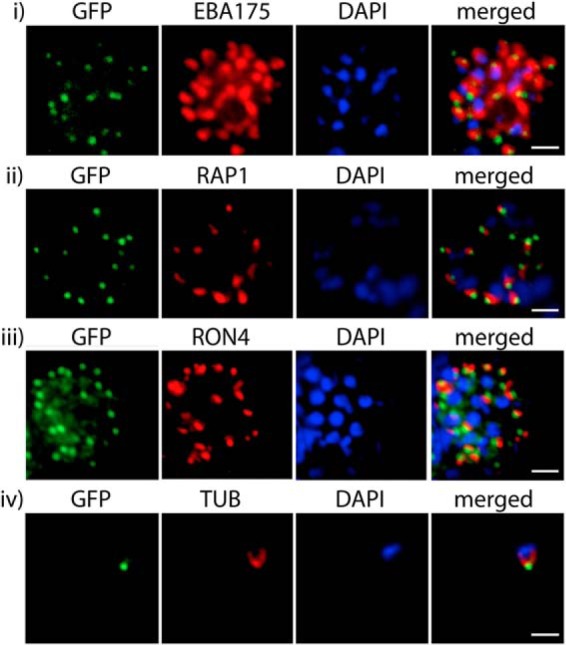
**PfMyoB-GFP is located at the apical pole of the merozoite.** Indirect immunofluorescence of PfMyoB-GFP (*green*) with various merozoite organelle markers (*red*), using antisera to *row i*, EBA175; *row ii*, RAP1; *row iii,* RON4; and *row iv,* α-tubulin (*TUB*). Samples were counterstained with DAPI (*blue*). The merged images are also shown. *Rows i–iii* show individual schizonts, and *row iv* shows an individual merozoite. *Scale bar,* 2 μm.

To establish more precisely the location of MyoB-GFP within the blood stage schizonts of *P. falciparum*, its position relative to protein markers of known apical structures such as the microneme and rhoptry organelles was examined using an indirect immunofluorescence assay (Fig. 5). These proteins included the microneme marker EBA175, the rhoptry bulb marker RAP1, the rhoptry neck marker RON4, and tubulin. Antibodies to GFP produced a compact discrete dot pattern of fluorescence located at the very apical end of the MyoB-GFP parasites, close to the localization of the apical markers EBA175, RON4, and RAP1. However, although in some cases the fluorescent signal partially overlapped with these markers, MyoB appeared to be in a distinct location within the cell. Examination of the localization suggested that the protein is anterior to the microneme marker, the rhoptry bulb, and even to the rhoptry neck. Partial colocalization with antibodies against tubulin in merozoites suggested that MyoB-GFP was coincident with or very close to the microtubule organizing center at the apical polar rings from which microtubules originate.

##### MyoB Does Not Relocate during Erythrocyte Invasion

**FIGURE 6.**
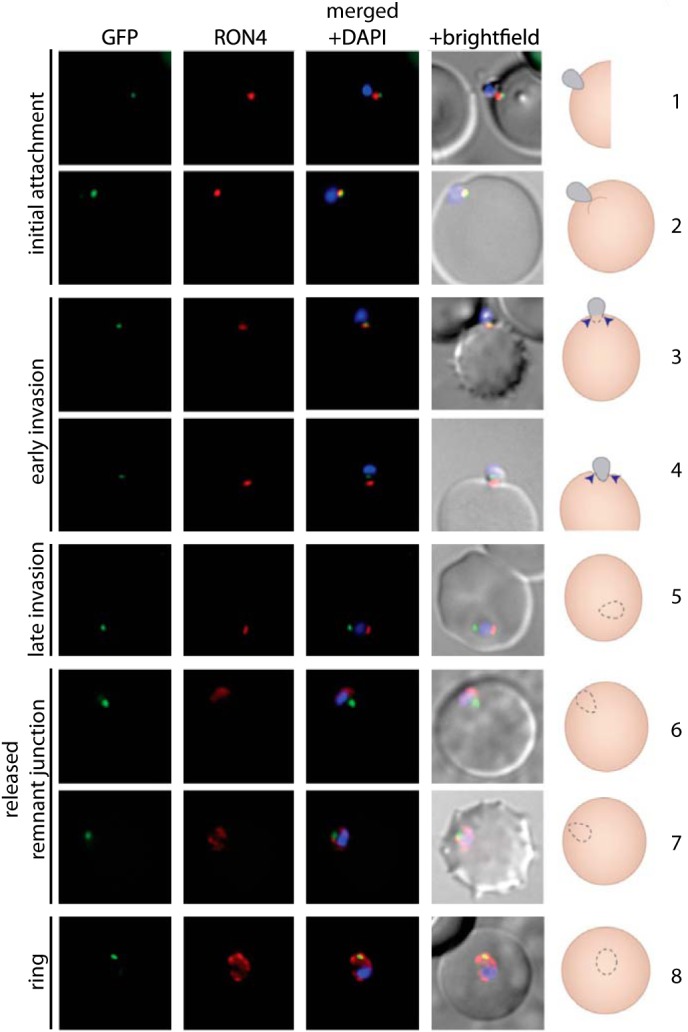
**PfMyoB-GFP remains at the anterior of the merozoite during invasion of the host cell.** MyoB-GFP-expressing *P. falciparum* parasites were fixed during various stages of invasion, and then MyoB-GFP (*green*) was revealed using rabbit anti-GFP antibodies, RON4 (*red*) was detected using mAb 24C6, and nuclei were stained with DAPI (*blue*). Merged images, including the differential interference contrast, are shown, as well as a schematic of the invasion stage in which the moving junction is shown by the *blue arrowheads*, the extracellular merozoite is *gray*, and the intracellular parasite is denoted by a *dotted line* and is uncolored. The invasion steps have been divided into initial attachment, followed by early and late stages of invasion as well as the final steps of invasion with the release of the remnant junction and formation of the ring stage. *Scale bar,* 2 μm.

The apical location of PfMyoB-GFP suggests that it might be involved in erythrocyte invasion in merozoites and perform a similar function in the other invasive stages within the life cycle. Therefore, we examined the location of the protein during invasion relative to RON4, which can be traced by indirect immunofluorescence assay from a single point close to the apex of the merozoite during attachment and through to completion of entry and formation of the parasitophorous vacuole (PV) (23). In free merozoites, PfMyoB-GFP is located at the anterior end of the cell, apical to RON4 (Fig. 6, *row 1*). At the initial attachment, there is a partial colocalization of PfMyoB-GFP with RON4 (Fig. 6, *row 2*). Then, as the merozoite starts the invasion process (Fig. 6, *rows 3* and *4*), RON4 is located anterior to PfMyoB-GFP, suggesting the secretion of RON4 from the rhoptry neck onto the host cell. As the parasite finishes invading the erythrocyte, RON4 is localized at the posterior of the parasite, and MyoB-GFP remains at the apical end of the cell (Fig. 6, *row 5*). RON4 then becomes delocalized in the remnants of the junction, which has closed the developing PV and plasma membrane and spreads in patches around the expanding PV membrane, suggesting loss of structural integrity of the junction at this stage, although PfMyoB-GFP remains as a single dot (Fig. 6, *rows 6* and *7*). During early ring stages, PfMyoB-GFP remains as a single dot located inside the PV membrane (Fig. 6, *row 8*). Because MyoB-GFP cannot be detected in trophozoite stages (see above), it is likely that after invasion has been completed PfMyoB-GFP is eventually degraded.

##### MyoB Does Not Associate with Components of the Glideosome Complex

Although MyoB is overall similar in size and domain structure to MyoA (1, 2), the localization data suggested that it occupies a very different compartment of the merozoite to that of MyoA. Nevertheless, these data do not exclude the possibility that it is associated with components of the glideosome, and therefore, we examined whether or not known glideosome proteins were bound to MyoB-GFP immunoprecipitated from parasite extracts. Mature *P. falciparum* schizont-stage parasites expressing MyoB-GFP were enriched and solubilized in buffer, and then proteins were immunoprecipitated with anti-GFP camelid nanobodies coupled to agarose beads. The precipitated proteins were then resolved by SDS-PAGE and subjected to Western blot analysis with antibodies specific to components of the glideosome. As controls, similar extracts were made from the parental 3D7 line and a parasite line expressing MyoA-GFP that had been produced in the same way (Fig. 1).

**FIGURE 7.**
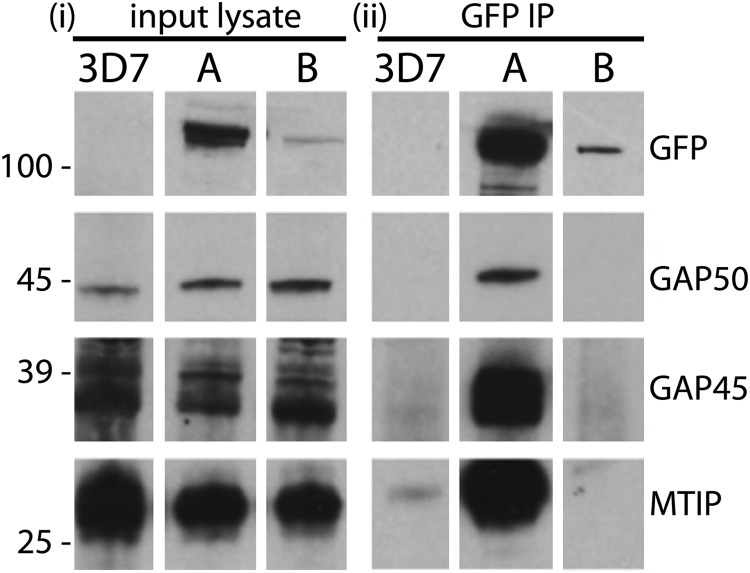
**PfMyoB-GFP does not associate with the glideosome components MTIP, GAP45, and GAP50.** *i,* Western blot of parasite lysates from 3D7, MyoA-GFP (*A*), and MyoB-GFP (*B*) parasite lines. *ii,* GFP-TRAP immunoprecipitates from corresponding parasite lysates (shown in *i*) separated by SDS-PAGE and probed with antibodies indicated on the *right* of each panel (rabbit anti-GFP, anti-GAP50, anti-GAP45, and anti-MTIP). Although GAP50, GAP45, and MTIP were present in all the lysates, they were detected in the MyoA-GFP immunoprecipitate but not in the MyoB-GFP immunoprecipitate. Molecular mass markers are indicated on the *left* in kDa.

A GFP-specific monoclonal antibody (mAb) detected bands corresponding to PfMyoA-GFP and PfMyoB-GFP (both ∼120 kDa) in the respective lysate and immunoprecipitate but not in lysates from 3D7 parasites (Fig. 7). The relative intensity of the bands in the two parasite samples indicated that PfMyoA-GFP is much more abundant than PfMyoB-GFP, as an equal amount of total parasite protein was loaded in each case. The MyoA-GFP immunoprecipitate contained MTIP, GAP45, and GAP50. However, none of these glideosome complex proteins were precipitated with PfMyoB-GFP, demonstrating that MyoB does not form a complex with known components of the MyoA glideosome.

##### Identification of a MyoB Protein Complex, Including a Putative Myosin Light Chain

To establish whether or not PfMyoB-GFP formed a complex with unknown proteins, the PfMyoB-GFP purified in the previous immunoprecipitation was resolved by SDS-PAGE, and proteins were identified following trypsin digestion and peptide analysis by LC-MS/MS. An identical immunoprecipitate from the lysate of wild-type 3D7 parasites was used as a control. A total of 11 proteins (each represented by more than two peptides) was identified in the PfMyoB-GFP precipitate and absent from the control (proteins are listed in Table 2). MyoB was represented by 40 peptides. None of the proteins from the glideosome complex (MyoA, MTIP, GAP45, and GAP50) were identified in this dataset, confirming the Western blot analysis of the precipitate presented in Fig. 7. Of the remaining 10 proteins, only two were represented by more than five peptides: PF3D7_1118700 (six peptides; 11.7% coverage), described as a “conserved *Plasmodium* protein of unknown function,” and Pf3D7_1211700 (seven peptides; 11.9% coverage), described as a putative minichromosome maintenance subunit 5. Although MCM5 seemed an unlikely MyoB partner because of its predicted location in the nucleus, PF3D7_1118700 encodes a predicted EF-hand containing domain at its C terminus, reminiscent of that present in other myosin light chains such as MTIP. The remaining proteins identified using this approach included polyubiquitin, SEC31, acyl-CoA synthetase, and a putative clathrin heavy chain.

**TABLE 2:**
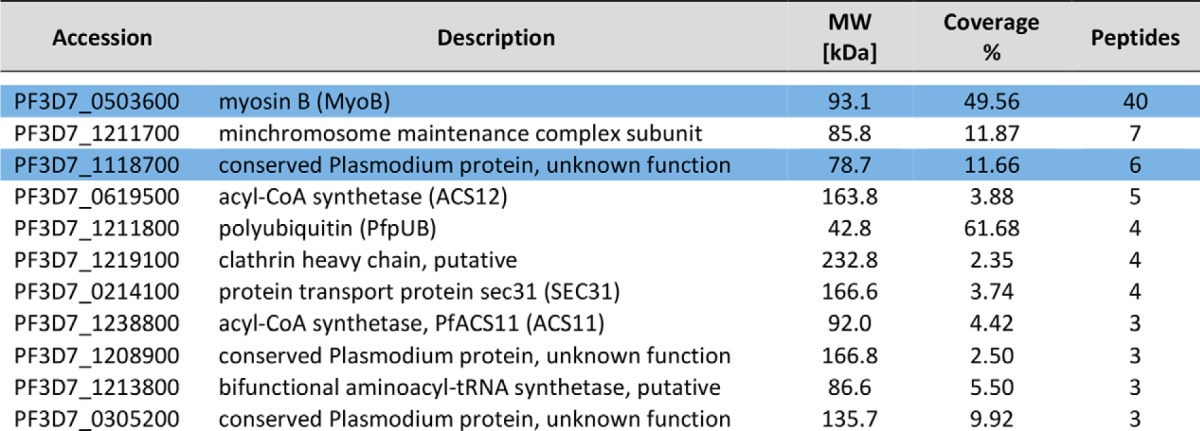
**Analysis of MyoB-GFP by mass spectrometry following immunoprecipitation from a parasite lysate** Each protein identified is listed with their PlasmoDB accession number, a description of the protein identified, and the molecular mass. The 4th column gives the percentage of the protein sequence covered by the identified peptides, and the number of unique peptides detected is in the 5th column. MyoB and its light chain are highlighted in blue.

**FIGURE 8.**
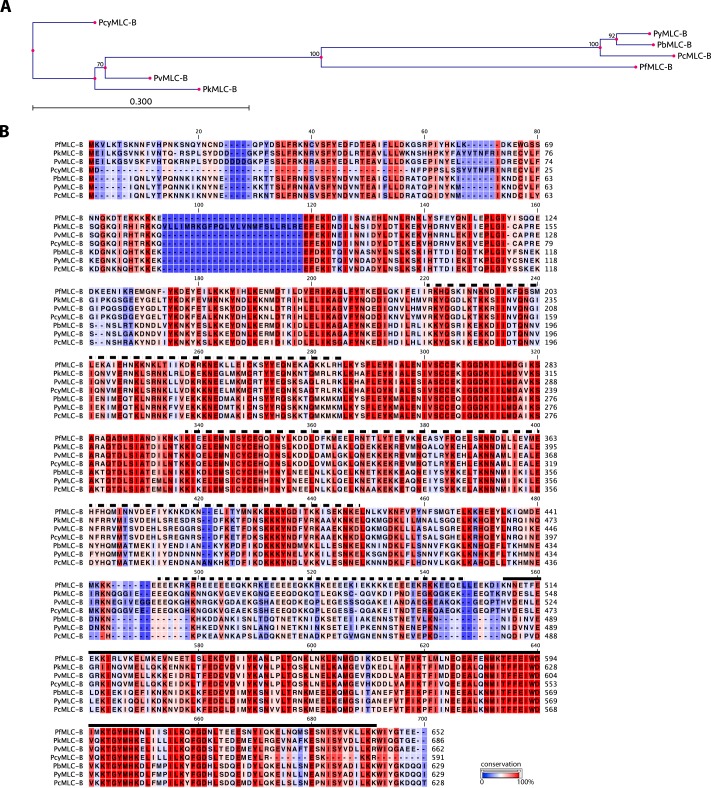
**Myosin light chain B phylogeny, structure, and expression.** *A,* phylogenetic tree with relationship between MLC-B sequences in *Plasmodium* species. The tree was prepared using the alignment shown in *B*. The neighbor-joining method was used, with 100 replicates used to generate bootstrap values, which are indicated at the branch points. The tree is drawn to scale, with branch lengths indicating the amount of variation (*scale bar,* 0.3 amino acid substitutions per site). *B,* alignment of *Plasmodium* MLC-B proteins. In PlasmoDB: *P. knowlesi* PkMLC-B, PKH_091610; *Plasmodium vivax* PvMLC-B, PVX_091570; *Plasmodium cynomolgi* PcyMLC, PCYB_092480; *P. berghei* PbMLC-B, PBANKA_092940; *Plasmodium. yoelii* PyMLC-B, PY17X_0931400; *Plasmodium chabaudi* PcMLC-B, PCHAS_091490. Regions of PfMLC-B predicted to form dimeric coiled-coil structures are marked with a *broken line above* the sequence. The calmodulin-like domain is marked with a *solid line above* the sequence.

**FIGURE 9.**
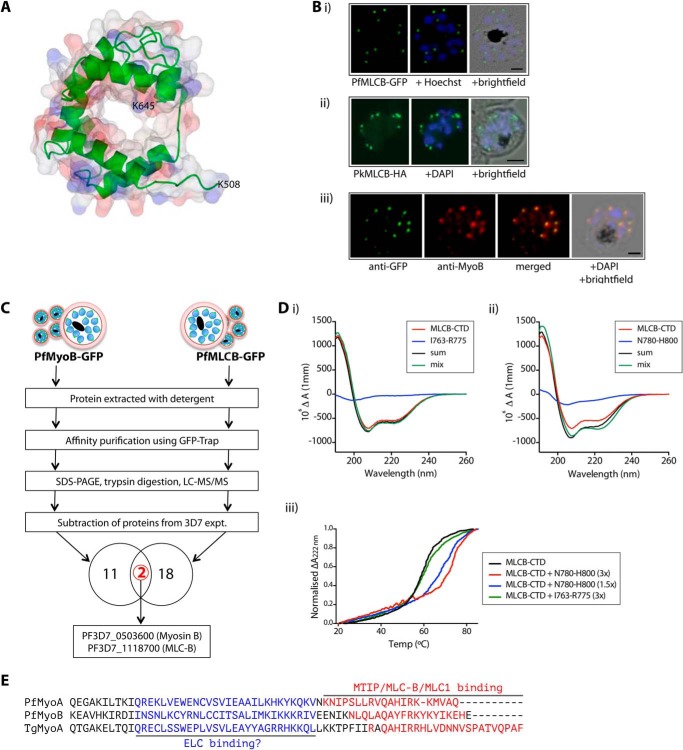
**PfMLC-B colocalizes in the cell with MyoB, binds to MyoB *in vivo,* and its C-terminal domain binds to the MyoB C-terminal sequence *in vitro*.** *A,* structural model of amino acids 508–645 of PfMLC-B. The protein backbone is shown as a *green ribbon*, with a space-fill model of the structure overlaid. *B, panel i,* MLC-B-GFP subcellular location in a *P. falciparum* schizont. GFP was detected by green fluorescence, and the nuclei (*blue*) were labeled with Hoechst dye prior to microscopic analysis. Merged images are also shown. *Scale bar,* 2 μm. *Panel ii,* MLC-HA localization to the apex of merozoites in a *P. knowlesi* schizont. The HA epitope was detected using a specific antibody, followed by a species-specific AlexaFluor 488-labeled secondary antibody. Nuclei (*blue*) were detected using DAPI. Merged images are also shown. *Scale bar,* 2 μm. *Panel iii,* indirect immunofluorescence of *P. falciparum* schizont to determine colocalization of MLC-B-GFP (*green*) with MyoB (*red*). Nuclei were counterstained with DAPI (*blue*). The merged images are also shown. *Scale bar,* 2 μm. *C,* analysis of proteins affinity-purified with GFP-Trap from lysates of parasites expressing either PfMyoB-GFP or PfMLC-B-GFP by SDS-PAGE, trypsin digestion, and LC-MS/MS. Following subtraction of the list of proteins detected in control experiments, the lists of proteins detected in the two preparations were compared. Two proteins, MyoB and MLC-B, were in common. *D,* analysis of the binding of the C-terminal domain of PfMLC-B to peptides derived from the sequence at the C terminus of MyoB by either circular dichroism (*panels i* and *ii*) or thermal unfolding in the presence of peptides based on the MyoB amino acid sequences, residues Ile^763^ to Arg^775^ and Asn^780^ to His^800^ (*panel iii*). *E,* alignment of the neck regions of PfMyoA, PfMyoB, and TgMyoA showing confirmed (*red*) and speculated (*blue*) light-chain binding regions.

The PF3D7_1118700 gene is 4.3 kb, with 18 exons, and in *P. falciparum* 3D7 asexual blood stages expression of the gene peaks in the late stage of schizogony, 41 h after invasion (33). The protein product is predicted to be 652 amino acids, with a molecular mass of 78.7 kDa; hereafter, we will refer to it as myosin light chain B (MLC-B). The protein is well conserved among all *Plasmodium* species for which sequence information is available (a phylogenetic tree and alignment of *Plasmodium* MLC-B proteins are shown in Fig. 8, *A* and *B*). The MLC-B C terminus (residues 508–645) resembles sequences from the EF-hand superfamily (Interpro: IPR011992) such as CaM and CaM-like calcium binding domains, which are also a feature of myosin light chains. We used the Phyre 2 protein fold recognition server (28) to create a structural model of the CaM-like domain of MLC-B. This shows a compact molecule that forms a structure with a channel through the center (Fig. 9*A*). We would expect the protein to form a clamp around an interacting peptide in an analogous manner to that described for CaM binding to the M13 peptide of myosin light chain kinase, the structure upon which this domain of MLC-B was modeled (34). Using the C-terminal CaM-like domain to perform BLAST analysis on the *Toxoplasma gondii* genome, most of the hits are small CaM or CaM-related proteins. One large protein of 885 amino acids was also identified, TGME49_250840, and annotated as a “hypothetical protein.” This protein is predicted to have the same overall domain structure as PfMLC-B, with a CaM-related domain at its C terminus and extensive predicted coiled-coil domains, and it has the attributes needed to be the orthologue of MLC-B in *T. gondii*.

##### MLC-B and MyoB Have the Same Subcellular Location and Form a Complex

To establish further whether or not the putative MLC-B is in a complex with MyoB in the cell, we first generated a *P. falciparum* parasite line that expresses MLC-B with GFP at its C terminus and a *P. knowlesi* parasite line that expresses MLC-B with a triple HA epitope tag at its C terminus (Fig. 3).

*P. falciparum* MLC-B-GFP was localized by live fluorescence microscopy to the extreme apex of merozoites (Fig. 9*B, panel i*), with expression restricted to those schizonts containing 10 or more nuclei, as was also the case for MyoB (Fig. 4*A*). Similarly, *P. knowlesi* MLC-B, identified by an antibody to the HA-tagged protein, could be clearly seen as a discrete dot at the very tip of the elongated merozoite within segmented schizonts (Fig. 9*B, panel ii*). Immunofluorescence showed a complete colocalization of MLC-B-GFP and MyoB in *P. falciparum* schizonts, with the proteins located at the apical tip of each merozoite (Fig. 9*B, panel iii*).

To confirm an *in vivo* interaction between MLC-B and MyoB, and to establish the identity of any other proteins that may bind to MLC-B, we immunoprecipitated MLC-B-GFP from extracts of late schizonts and analyzed the precipitated proteins by LC-MS/MS using identical methodology as for PfMyoB-GFP. A list of proteins present in the MLC-B-GFP immunoprecipitate but absent in a control 3D7 precipitate was constructed (Table 3) and compared with the MyoB-GFP dataset (Table 2). Only two proteins were present in both the MLC-B-GFP and MyoB-GFP precipitates, MyoB and MLC-B (Fig. 9*C*). Overall, these data indicate that MLC-B and MyoB have the same subcellular location and form a protein complex within the parasite, consistent with MLC-B being a myosin light chain specific for MyoB.

**TABLE 3:**
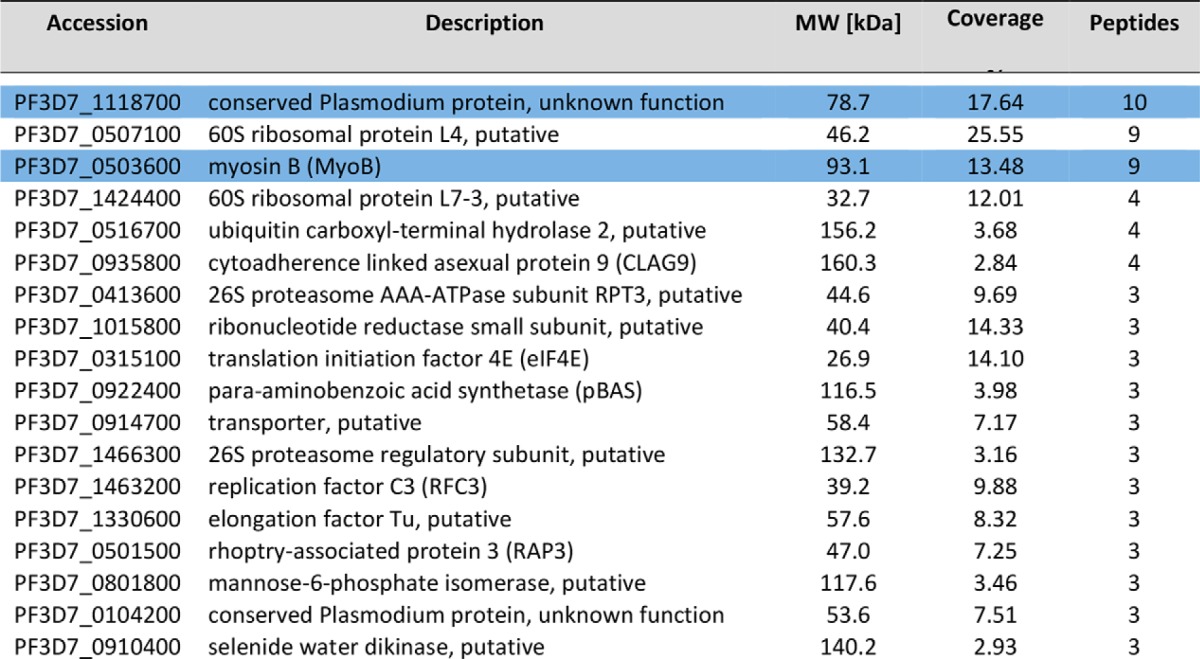
**Analysis of MLC-B-GFP by mass spectrometry following immunoprecipitation from a parasite lysate** Each protein identified is listed with their PlasmoDB accession number, a description of the protein identified, and the molecular mass. The 4th column gives the percentage of the protein sequence covered by the identified peptides, and the number of unique peptides detected is in the 5th column. MLC-B and MyoB are highlighted in *blue*.

##### Calmodulin-like Region of MLC-B Binds to the C Terminus of MyoB

The similarity of the C terminus of MLC-B to the EF-hand superfamily suggested that the interaction between MLC-B and MyoB may be mediated by binding of the calmodulin-like C-terminal domain (CTD) of MLC-B to the neck region of MyoB. This is analogous to the interaction between MTIP and MyoA (7, 35).

To study the MLC-B CTD in more detail, we expressed the C-terminal 156 amino acids, (residues 497–652) in *Escherichia coli* and purified the protein to homogeneity by affinity chromatography. Initial analysis of the overall structure of the protein was performed using far-UV CD (Fig. 9*D, panels i* and *ii*, *red line*). Comparing the spectrum of MLC-B CTD with those of reference proteins predicted that it contained 40% α-helix, 14% β-sheet, 17% turn, and 27% unordered structure. There was no change in the CD spectrum upon the addition of calcium to 1 mm (data not shown), indicating that an interaction with calcium was unlikely.

We next tested the ability of MLC-B CTD to bind to peptides from the C terminus of MyoB. Using far-UV CD, we looked for changes in the spectrum of MLC-B and peptide that may occur upon binding between the two. Fig. 9*D, panels i* and *ii,* shows the overlaid spectra of MLC-B CTD (*red*), the peptide alone (blue), the calculated sum of the MLC-B CTD and peptide spectra (*black*), and a solution of MLC-B CTD and peptide (*green*). For a control peptide that spans residues Ile^763^ to Arg^775^ of MyoB, the peptide spectrum is characteristic of a random coil, and there is no difference between the sum of the MLC-B CTD and peptide spectra and that of a solution containing the two components (Fig. 9*D, panel i*). This suggests that there is no interaction between the two, although there is the possibility that they interact without an accompanying structural change. Fig. 9*D, panel ii,* shows the same analysis for a peptide from the extreme C terminus of MyoB. The solution of MLC-B CTD and Asn^780^ to His^800^ peptide (Fig. 9*D, green*) showed a different spectrum when compared with the sum of the individual spectra (*black*), indicating that they interact and that there is an increase in α-helix content in one or both of the components upon binding.

The interaction of MLC-B CTD with the MyoB Asn^780^ to His^800^ peptide was confirmed by performing thermal unfolding experiments. A solution of MLC-B CTD with or without peptide was subjected to progressively higher temperatures, and the change in differential absorbance at 222 nm was measured to track the transition of MLC-B from a folded to an unfolded state. The presence of a strong binding partner would be expected to stabilize a protein, necessitating an increased input of energy for this transition to occur. Fig. 9*D, panel iii,* shows the thermal denaturation profile of MLC-B CTD alone (in *black*) or in the presence of MyoB C-terminal peptides as follows: a 3-fold molar excess of the Ile^763^ to Arg^775^ peptide (in *green*), and a 1.5- or 3-fold molar excess of Asn^780^ to His^800^ peptide (*blue* and *red,* respectively). The presence of the Ile^763^ to Arg^775^ peptide had no effect on the thermal denaturation of MLC-B CTD. In contrast, in the presence of a 1.5-fold molar excess of the Asn^780^ to His^800^ peptide, the melting temperature (*T_m_*) of MLC-B CTD increased from 59 to 67 °C. With a 3-fold molar excess of the Asn^780^ to His^800^ peptide, the *T_m_* increased to 70 °C. These results indicate a strong interaction between MLC-B CTD and the Asn^780^ to His^800^ peptide.

Fig. 9*E* shows an alignment of the neck regions of PfMyoA, PfMyoB, and TgMyoA. The regions at the C terminus where MTIP, MLC-B, and MLC1 bind are highlighted (although for TgMyoA the binding has been inferred rather than directly shown; deletion of this region causes mislocalization of MyoA in *T. gondii* tachyzoites (36)). All three binding regions contain a preponderance of basic residues that for both MyoA proteins have been shown to be essential determinants of binding (6, 36). It seems likely that basic residues in the MLC-B binding region of MyoB will also be important determinants of binding. Recently, it has been demonstrated that the TgMyoA motor complex contains an additional light chain, designated TgELC (37). It has been speculated that binding of the additional light chain takes place N-terminal to the binding site of MLC1 (3), and indeed it has been demonstrated that both light chains are required in order for TgMyoA to achieve the maximal movement of actin (38). All three of the class XIV myosins shown in Fig. 9*E* have a similar amount of “free” space at the appropriate position. It is likely that there is an additional light chain that binds to this position in MyoB, although we did not detect a suitable candidate in our immunoprecipitation studies (Tables 2 and 3), and due to the degeneracy of the IQ motifs in apicomplexan myosins, it is not possible to speculate as to the precise binding site.

##### Coiled-coil Region of MLC-B

**FIGURE 10.**
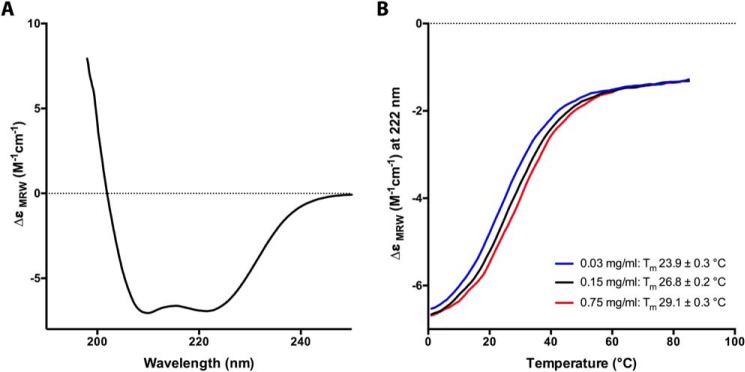
**PfMLC-B(301–409) is highly helical and shows concentration-dependent thermal unfolding.** *A,* far-UV CD spectrum of a solution containing 0.15 mg/ml PfMLC-B(301–409). The spectrum is characteristic of an extremely α-helical protein. *B,* thermal denaturation of PfMLC-B(301–409) performed at three protein concentrations: 0.03, 0.15, and 0.75 mg/ml. Unfolding was monitored by observing the change in the CD signal at 222 nm. The *T_m_* of the protein increased with concentration, consistent with a nonmonomeric protein.

The N-terminal remainder of MLC-B is predicted to be largely α-helical. By *in silico* analysis using Scorer (27) with the threshold for coiled-coil prediction set to 99%, three regions of PfMLC-B were predicted to form dimeric coiled-coils: regions 184–249, 301–409, and 446–501. All three were expressed as GST fusion proteins in *E. coli*, but only the largest of the three was soluble and therefore suitable for further analysis (the 109 amino acid region from 301–409). After removal of the GST moiety by proteolysis, far-UV CD spectroscopy was used to assess the secondary structural content of the recombinant protein (Fig. 10*A*). Analysis of the spectrum using standard methods (32) shows that the protein is very highly helical, *i.e.* composed of 70.7% α-helix, 2.3% β-sheet, 11.7% turn, and 15.4% random coil. This extremely high helicity is consistent with the prediction that the protein adopts a coiled-coil conformation. We next assessed the behavior of the protein at different concentrations in thermal unfolding experiments, monitoring the unfolding by measuring the CD signal at 222 nm. We analyzed solutions of PfMLC-B(301–409) at three different concentrations, covering a 25-fold range in protein concentration. The *T_m_* of PfMLC-B(301–409) increased with concentration (Fig. 10*B*). Thermal unfolding of a monomeric protein should be independent of the protein concentration, whereas in dimeric or trimeric proteins, the *T_m_* will increase with increasing protein concentration (32). We can therefore confidently state that the region of PfMLC-B from 301 to 409 adopts a highly helical dimeric (or trimeric) structure that is consistent with a coiled coil.

## DISCUSSION

We have characterized a novel class XIV myosin, MyoB in *Plasmodium*. Like the other short class XIV myosin, MyoA, this myosin has no tail region; the catalytic head domain is followed by a modified neck region, which for MyoA is the binding site for a light chain that has been designated MTIP. Whereas MyoA is part of the glideosome machinery that powers parasite motility and invasion, the location of MyoB in the cell was uncertain (15). To characterize MyoB, we used three parasite species, *P. falciparum*, *P. knowlesi,* and *P. berghei*, taking advantage of the unique experimental opportunities they each provide. *P. berghei* allows stages of the life cycle in the mosquito and in the liver to be analyzed, and there are robust systems for genetic manipulation of the parasite. *P. knowlesi* has been recently adapted to growth *in vitro* in human red cells; transfection is highly efficient, and the merozoites are significantly larger than those of *P. falciparum* and *P. falciparum* for which growth *in vitro* can be efficiently synchronized, and there are many reagents to identify specific proteins. We show here that the *myob* gene is expressed in the motile and invasive stages of the parasite's life cycle: merozoite, ookinete, and sporozoite forms. By using tagged MyoB, we show that the protein locates to a compact and discrete apical region at the very tip of these three polarized cells. This location is distinct from that of MyoA, which is detected around the periphery of the parasite (39). During the development of asexual blood stages of *P. falciparum*, the protein is only detected late in the cycle, when there are 10 or more nuclei, and just before cytokinesis (segmentation) occurs to form individual merozoites.

Colocalization studies in merozoites, using rhoptry, rhoptry neck and micronemal markers confirmed the localization of MyoB-GFP to the extreme apex of merozoites, anterior to all of the organelle markers used. We also demonstrated that its location partially overlapped with α-tubulin. Tubulin forms the two or three subpellicular microtubules known in merozoites as f-MAST, anchored at the anterior polar ring and running in parallel along the merozoite periphery toward the posterior of the merozoite (40). The localization of microtubules has been shown in previous studies by immunofluorescence (40, 41) and electron microscopy (42). It is possible that MyoB is located at the polar ring(s); however, definitive localization studies will require immunoelectron microscopy. We have been unsuccessful in this approach with both GFP- and HA-tagged parasite lines, perhaps due to the very low abundance of the protein. It should be noted that in *P. berghei* merozoites, ookinetes, and sporozoites in which actin filaments have been stabilized using jasplakinolide, F-actin is detected by an antibody specific for actin I at the apical end and periphery of the cells, suggesting that these are sites of high actin turnover (43). We have demonstrated that MyoB is present at the apical ends of these cells, consistent with MyoB being a motor that moves filaments of actin I.

The location of MyoB at the apex of the three invasive stages of the parasite life cycle suggested that it has a role in cellular invasion. Invasion is a complex process in which reversible initial attachment is followed by the elaboration of a moving junction between parasite and host cell that moves backwards over the parasite surface as it enters the RBC. The moving junction can be visualized by microscopy with antibodies to the rhoptry neck protein RON4. We examined the fate of MyoB during the process of merozoite invasion of red blood cells and compared it with RON4. Although initially anterior to RON4, MyoB remained static as RON4 moved to the apex of the merozoite and then backwards to the rear of the cell as part of the moving junction. During this invasion process, MyoB-GFP remained at the apical tip of the merozoite, did not participate in the moving junction, and was still apical in newly invaded ring stages until it finally disappeared. Although its precise function remains unknown, it is possible that MyoB has a role in formation of the apical polarity of the cell or a structural/mechanical role in secretory vesicle fusion during the invasion process, consistent with its static localization. Of course, it would be of great interest to study the effects of deletion of the *myob* and/or *mlc-b* genes on parasite development. We have attempted to knock out *mlc-b* in both *P. falciparum* and *P. berghei* without success. This suggests that the MyoB·MLC-B complex is likely to be essential for parasite survival. Experiments are underway to confirm these data with *myob*. If the complex is confirmed to be essential, alternative strategies, for example inducible gene knockouts, will have to be employed to study the consequences of gene deletions.

Because of the overall predicted structural similarity between MyoB and MyoA, it was of interest to establish whether or not MyoB interacted with MTIP or other components of the glideosome complex. MTIP (MLC1 in *T. gondii*) had been identified as one of the light chains for MyoA, and the association occurred through the degenerate IQ motif at the extreme C-terminal end of MyoA (6, 8, 9, 44). Related sequences are also present at the C terminus of MyoB. Analysis of immunoprecipitated MyoB-GFP by Western blotting with protein-specific antibodies provided no evidence for an association of MTIP, GAP45, or GAP50 with MyoB, further confirmed by mass spectrometry analysis. In these experiments, we identified a novel light chain for MyoB, designated MLC-B. MLC-B was shown to be localized to the apex of merozoites, at a location indistinguishable from MyoB. MLC-B is an unexpectedly large protein, composed of two parts as follows: the EF-hand containing calmodulin-like CTD and the much larger N-terminal domain. We have demonstrated that the CaM-like CTD of MLC-B is able to bind a peptide from the extreme C terminus of MyoB. Like the CaM-like domain of MTIP, this domain in MLC-B does not possess any of the specific residues likely to be important in the coordination of calcium ions by EF-hands (45). Therefore, although there may be a structural similarity to calmodulin in this region, it is unlikely that calcium plays a role in its function or structure.

MLC-B is the largest myosin light chain to be identified in any species, and its structure is unique. Light chains are known to be required for full functioning of myosin motors (38, 46); however, most are CaM-like molecules with molecular masses less than 20 kDa (47). MTIP has an N-terminal extension that has been demonstrated to be important for its interaction with GAP45, another member of the glideosome complex (13); however the N-terminal extension of MLC-B is far larger, comprising more than 75% of the MLC-B sequence. The N-terminal region of MLC-B is predicted to primarily contain α-helical structural elements, and *in silico* analysis of this sequence suggested that it would form a dimeric coiled coil. We have shown by CD and thermal denaturation experiments that a region of the predicted coiled coil of PfMLC-B is indeed highly helical and exhibits concentration-dependent thermal unfolding. These characteristics are consistent with this region forming a coiled-coil structure, although we cannot distinguish between dimeric and trimeric coiled coils using these methods. It may be that dimerization of this unusual light chain is the way that the small tailless MyoB achieves functionality and/or correct localization. To generate movement, intrinsically monomeric myosins require either dimerization or anchoring into a fixed structure to form an array. For example, the apicomplexan class XIV myosin MyoA is anchored into the parasite's inner membrane complex by virtue of membrane interactions by its associated molecules, including the light chain MTIP (13, 14, 48), and dimerization of myosin V (49) and myosin VI (50) is required for their functions. It may be that the smaller than usual class XIV myosins make up for their lack of tail by having larger than usual light chains that are able to functionally compensate. Further work to address the regulation, regions involved in targeting to its apical location, and further possible binding partners of the MyoB·MLC-B complex will be crucial to address this question, which is fundamental to identifying the role of MyoB.

## Footnotes

6 E. Knuepfer and A. A. Holder, unpublished data.

## Footnotes

5 The abbreviations used are:

Myo: myosin
GAP: glideosome-associated protein
MLC-B: myosin B light chain
CaM: calmodulin
CTD: C-terminal domain
PV: parasitophorous vacuole
IFA: indirect immunofluorescence assay
BisTris: 2-[bis(2-hydroxyethyl)amino]-2-(hydroxymethyl)propane-1,3-diol
MTIP: myosin A tail domain-interacting protein.
